# Challenges and strategies in conducting sexual and reproductive health research among Rohingya refugees in Cox’s Bazar, Bangladesh

**DOI:** 10.1186/s13031-020-00329-2

**Published:** 2020-12-01

**Authors:** Rushdia Ahmed, Bachera Aktar, Nadia Farnaz, Pushpita Ray, Abdul Awal, Raafat Hassan, Sharid Bin Shafique, Md Tanvir Hasan, Zahidul Quayyum, Mohira Babaeva Jafarovna, Loulou Hassan Kobeissi, Khalid El Tahir, Balwinder Singh Chawla, Sabina Faiz Rashid

**Affiliations:** 1 BRAC James P Grant School of Public Health, BRAC University, 5th Floor, (Level-6), icddrb Building, 68, Shaheed Tajuddin Ahmed Sarani, Mohakhali, Dhaka, 1212 Bangladesh; 2 Department of Reproductive Health Research, World Health Organization, Geneva, Switzerland; 3 Health Sector Coordination Office, World Health Organization, Cox’s Bazar, Bangladesh

**Keywords:** Rohingya refugees, Humanitarian crises, Sexual and reproductive health, Situation analysis, Adolescent girls, Women

## Abstract

**Background:**

Rohingya diaspora or Forcibly Displaced Myanmar Nationals (FDMNs), took shelter in the refugee camps of Cox’s Bazar, Bangladesh due to armed conflict in the Rakhine state of Myanmar. In such humanitarian crises, delivering sexual and reproductive health (SRH) services is critical for better health outcomes of this most-at-risk population where more than half are adolescent girls and women. This is a reflective paper on challenges and related mitigation strategies to conduct SRH research among FDMNs. The research on which this paper is based employed a concurrent mixed-method design combining a cross-sectional survey and qualitative interviews and group discussions with FDMNs to understand their SRH needs and demand-side barriers. Assessment of health facilities and qualitative interviews with healthcare providers and key stakeholders were carried out to assess facility readiness and supply-side barriers.

**Challenges and strategies:**

The researchers faced different challenges while conducting this study due to the unique characteristics of the FDMN population and the location of the refugee camps. The three key challenges researchers encountered include: sensitivity regarding SRH in the FDMNs, identifying appropriate sampling strategies, and community trust issues. The key approaches to overcome these challenges involved: actively engaging community members and gatekeepers in the data collection process to access respondents, identifying sensitive SRH issues through survey and exploring in-depth during qualitative interviews; and contextually modifying the sampling strategy.

**Conclusion:**

Contextual adaptation of research methods and involving community and local key stakeholders in data collection are the key lessons learnt from this study. Another important lesson was researchers’ identity and positionality as a member of the host country may create distrust and suspicion among the refugees. The multi-level complexities of humanitarian settings may introduce unforeseen challenges and interrupt research plans at different stages of research which require timely and contextual adaptations.

## Background

During humanitarian crisis, more than half of the displaced population is comprised of women and children as reported by the United Nations Population Fund (UNFPA) [1]. Displaced and refugee women worldwide mostly remain at their reproductive age [1]. Rape, sexual abuse, human-trafficking and violence are used as tactics of war, making women and girls the most-vulnerable and most-at-risk group during wars and conflicts [2]. They suffer immensely as reproductive health services including prenatal care, assisted delivery and emergency obstetric care is scarce but in high demand [3]. Many refugee women also lose access to family planning services, exposing them to unwanted pregnancies [4, 5]. In precarious conditions such as the humanitarian emergency, managing sexual and reproductive health (SRH) disparities for the affected, specifically for women and adolescent girls is critical for better health outcomes and quality of life [6, 7].

Bangladesh has been affected by the global refugee crisis as a country hosting around a million Forcibly Displaced Myanmar Nationals (FDMNs) [8]. Armed conflict in the Rakhine state on August 2017 triggered a massive exodus of Rohingyas to Bangladesh, the geographically proximate country, which has created a unique humanitarian crisis condition. More than 855,000 Rohingya people have fled to Cox’s Bazar, the southeast coastal district of Bangladesh, since then [9]. This Rohingya diaspora have settled in the collective sites^1^ (84%), collective sites with host communities^2^ (12%), and dispersed sites^3^ in host communities (4%) [10]. The arrival of this large population has created complex socio-cultural, economic and political impacts on the country’s health and development sectors including the local host community in terms of managing the basic needs, health, wellbeing, jobs, education and moreover, social cohesion of these traumatized populations [11, 12]. The influx of large numbers of FDMNs also creates an imbalance in the demand and supply chain markets [11, 12]. In response to such challenges in the Rohingya refugee camps in Cox’s Bazar, the development sector, both national and international, was galvanized into action, with support from Bangladesh’s people and government [10].

In spite of the interventions of many national and international organizations, the FDMNs, especially adolescent girls and women face many challenges including human trafficking, rape, prostitution [12]. Being at their reproductive age, they are also in need SRH services including pregnancy and delivery care, family planning services, menstrual health, safe abortion etc. However, their SRH care seeking behavior is highly influenced by the orthodox and conservative religious and cultural values, and the continuous deprivation of services that the FDMNs experienced in their own country in Myanmar [13, 14]. The scale of influx into Cox’s Bazar district and the scarcity of resources resulted in a critical humanitarian emergency that exceeded the coping capacity of the local administration and health systems, which impacts the already congested health response. Therefore, management of sexual reproductive health (SRH) disparities among women as a result of humanitarian crises requires thoughtful and innovative SRH specific service delivery packages and the generation of evidence to inform programme design, implementation and practice [10]. This reflective paper aims to describe the research challenges in conducting the SRH study with FDMNs and strategies utilized to overcome those challenges.

## Research study

The research study on which the present analysis of research challenges is based was part of a multi-country project of the World Health Organization (WHO), which intends to deliver integrated comprehensive sexual and reproductive health and rights (SRHR) services to meet the immediate SRHR needs of extremely vulnerable adolescent girls and women in acute humanitarian crises in three countries – Bangladesh (Cox’s Bazar), the Democratic Republic of the Congo (Kasai) and Yemen. In Bangladesh, BRAC James P Grant School of Public Health at BRAC University conducted the original research with an aim to understand SRH needs, service seeking behavior and service utilization related barriers (both demand and supply side) among the FDMN adolescent girls and women residing in the Rohingya refugee camps in Cox’s Bazar, Bangladesh. It also examined the readiness of health facilities to meet SRH needs of this population. This attempted to provide evidence to the original project for designing and delivering a comprehensive SRH service package to this vulnerable segment of FDMNs.

The original study was conducted in 10 randomly selected Rohingya refugee camps in Ukhiya and Teknaf sub-districts of Cox’s Bazar district using a concurrent mixed methods approach. Data was collected from August – November 2018. A cross-sectional household survey and in-depth interviews (IDIs) with adolescent girls and adult women aged 12–59 years along with IDIs and focus group discussions (FGDs) with broad range of community stakeholders (adult males, influential community members such as local leaders like Majhiis^4^ and religious leaders like Imams, and informal providers) were conducted to understand SRH needs and demand-side barriers. Health facility readiness for providing comprehensive SRH services at different levels was also assessed through a facility assessment survey designed by the research team by adapting WHO Service Availability and Readiness Assessment (SARA) tool [15]. IDIs with formal healthcare providers, and Key Informant Interviews (KIIs) with a range of key stakeholders and implementers including management and decision-making level staff from government, national and international non-governmental organizations (NGOs) and UN agencies were carried out to understand the supply-side barriers in providing SRH services to FDMNs. Several adjustments were made to the study design during implementation of the research due to the unique situation in the crisis setting that will be described in the main section on *Challenges to research and strategies utilized*. A detailed description of the study design employed in the original research is reported in Ahmed et al. 2019 [16].

## Scientific importance of this research

Implementing comprehensive SRH services to FDMNs requires evidence on traditional practices, local indigenous cultural health and illness models and comprehensive understanding of barriers and existing vulnerabilities [17, 18]. Credible information on the SRH of the FDMNs from their home countries are also important to consider, so are their behavior and practices in accessing these services. In Myanmar, they are mostly deprived of SRH services, counselling and information due to unavailability as they grow up isolated in a closed community setting with little access to equitable and quality SRH-related care, ongoing discrimination, violence, persecution, and their cultural and religious conservativeness overall as a community [19]. As hundreds and thousands of them have fled to neighboring countries like Thailand and Bangladesh, managing their reproductive health and quality of life has always remained a challenge [20].

In the Rohingya refugee camps of Cox’s Bazar, more than half (52%) of the FDMN population comprises adolescent girls and women who are even more vulnerable in the humanitarian context [12, 17]. The risk of morbidity and mortality related to pregnancy, childbirth, sexual exploitation, violence and diseases is higher for them in such complex emergencies. Literature suggest, there is a strong cultural phenomenon of child marriage among the FDMNs [21]. For adolescent girls, menstrual hygiene and related practices are of interest for their better SRH outcomes, however, insufficient access to safe facilities prevail [22]. Another important SRH-related service lacks attention and evidence – the abortion care [23]. Systematic sexual violence, unintended and unwanted pregnancies make the need for abortion care greater [23], however, extremely opposed by community religious leaders and influential, resulting in unsafe abortions [19]. Although some organizations are providing the “Minimum Initial Service Package (MISP) of SRH”,^5^ access to these essential reproductive and maternal health services remains a major concern [12, 24], especially in the new settlements and the hardest-to-reach camps. One of the objectives of MISP is to plan for expanding services to comprehensive SRH services^6^ once the situation becomes stable. Therefore, after 1 year of the recent influx, the Health Sector and SRHR sub-sector has taken initiatives to introduce a comprehensive SRH service package in the refugee camps of Cox’s Bazar. To attain the target of more than 55% institutional delivery by the Joint Response Plan 2019, considerable efforts and better understanding of both demand and supply side barriers is required [25]. Evidence is critical to improve the SRH priorities and needs of adolescent girls and women living in the camps. It is significant to assess the SRH gaps of FDMNs as there remains knowledge gap. Conducting research in such crises and a protracted humanitarian setting is critical and timely, and provides a better understanding of the situation, which will help humanitarian aid agencies effectively design and implement comprehensive SRH service package for FDMNs. Addressing the SRH gaps will help in increasing access to life-saving services and begin to build a foundation for meeting the SRH needs for FDMNs. Efforts have been made by continuous stakeholder engagement with the Ministry of Health and Family Welfare, Government of Bangladesh and the Health sector headed by WHO and SRH sub-sector by UNFPA since inception of the study, to ensure that programme and local political actors are aware of the SRH service provision situation identified through this research.

## Challenges to research and strategies utilized

The researchers faced different challenges while conducting this study due to the unique characteristics of the FDMN population and the location of the refugee camps.

### Cultural sensitivity of SRH issues

Culturally, SRH issues are considered as issues of shame and unacceptable to be discussed openly in many countries due to socio-cultural and religious norms and attitudes resulting in stigma around the concept of SRH [26, 27]. The FDMN community is no different. Their health related perceptions, knowledge and practices are predominated by cultural and religious beliefs due to decades of discrimination and deprivation from access to formal SRH education and services in Myanmar [28].

#### Challenges

Given the sensitive nature of SRH topics in the conservative culture of the FDMN community, asking questions during interviews, especially issues on menstrual health, family planning, sexually transmitted infections (STIs) and abortion, were a big challenge. Adolescent girls and women were reluctant and shy to talk about their experiences. It was indeed challenging to collect information on STIs due to the sensitivity and stigma associated with the issue because of the moral connotations. No biomedical tests were performed in the original study to identify STI symptoms among any respondents due to resource constraints. In the quantitative survey, data was collected only on symptoms (reported by the respondents) during interviews. However, because of the shame and embarrassment around sharing such symptoms with outsiders, very limited information was found on STI symptoms, service availability and health care seeking behavior among FDMN adolescent girls and women. Although the risk of STIs and transmission of HIV among the Rohingyas migrated to Bangladesh, were reportedly higher [29], 62% respondents mentioned no STIs symptoms in our study [30].

Abortion was another sensitive topic that women felt uncomfortable discussing due to socio-cultural and religious beliefs, stigma and fear of being socially isolated if such incidents were unveiled. A total of only four women (three identified during the survey and one during IDIs with women) reported having an abortion in their lifetime after migrating to the Cox’s Bazar refugee camps. During pre-testing of the survey questionnaire, the research team tried to ask family planning and abortion related questions to unmarried adolescent girls, but had to change the plan due to discomfort and high resistance from their parents.

Interviewing adolescent girls was more challenging in comparison to adult women. At the beginning of the survey, the research team attempted to interview one adult woman and one adolescent girl from the same household, simultaneously. However, because of the cultural reservations on discussing SRH issues with adolescents, especially who are unmarried [14], parents were more protective and reluctant to allow their teenage daughters to talk about SRH issues with the interviewers in the quantitative survey. Even after giving parental consent, the mothers of the selected adolescent girls wanted to accompany their daughters during the interview with an intention to monitor that sensitive issues are not discussed with the interviewers. Therefore, in most of the cases, adolescent girls felt uncomfortable even responding to questions on menstrual health and hygiene, and preferred to keep silent resulting in discontinuation of the interview.

Several times, male family members were present at home during data collection due to restricted access to income generation activities. Their presence made female respondents uncomfortable to speak openly about their SRH related experiences, though many of them wanted to share. It was even harder to communicate with the male members of this community, who had the tendency to avoid such discussions by referring to the research topics as “female issues” and hesitated to discuss SRH issues due to their conservative views. Though male facilitators conducted the FGDs, some respondents felt uncomfortable even after building rapport and left the discussion just after the introduction of SRH related topics.

#### Strategies employed

Due to the cultural sensitiveness, questions on STIs, abortion and family planning, were only asked to married adolescent girls and women. This was explored further during IDIs. Only menstrual hygiene related issues were discussed with unmarried adolescent girls.

The research team visited the selected block of a selected camp at least for three consecutive days to collect required numbers of samples for the survey. During those visits, rapport was built with the community members and key gatekeepers. The female members of research team members accompanied data collectors to the selected households during survey data collection, and had informal discussions with respondents before and after data collection with an aim to build rapport and select potential respondents for qualitative interviews (IDIs). After the preliminary selection of a potential respondent for IDI, one/two female members of the research team visited that household, had informal discussions again and invited the respondent to participate in IDI. As the respondent participated in the survey, she was familiar with the research topics. Therefore, it was comparatively easier to purposively select respondents who were willing to participate in IDIs to share their experiences in detail. This also allowed for gradual approach in asking sensitive SRH-related questions and their lived experiences [31].

To create a comfortable environment for the adolescent girl respondents and persuade their parents, the survey data collection strategy was modified. At first, the selected adult woman of a household was interviewed (in most cases, mothers having adolescent girls). Later, she was approached about interviewing her adolescent daughter. This strategy proved effective as the mother was already informed about the type of questions, which gave an assurance that no culturally sensitive issues would be discussed with her daughter. In some cases, mothers requested interviewers to discuss menstrual health related issues with their daughters for enhancing their knowledge.

To give female respondents a more favorable environment to talk about SRH issues, the male members of the research team spoke with the male family members about the study topics and the importance of research, and kept them busy outside home by discussing general issues of their lives in the camp. Those informal discussions also gave some insights into the lives of FDMNs and their cultural systems, which was not captured through other data collection methods.

The research team adopted a strategy to attract the male respondents to continue FGDs till completion. They trained male data collectors in techniques to persuade male community members and orient a particular participant on the research topics prior to conducting FGDs. That particular participant acted as a bridge between the community and the research team during discussions. These particular participants helped in engaging discussions by reiterating that female SRH as a topic is equally important for male members as they make key decisions regarding female SRH issues and healthcare seeking. This strategy was very helpful for successful completion of FGDs and collecting in-depth information.

### Methodological challenges

While conducting research in a humanitarian context, it is important for the research team to be flexible and innovative with the methodologies and test the feasibility of the planned strategy before starting the actual data collection.

#### Challenges

For the quantitative survey, we followed guidelines prepared by UNFPA and Save the Children for SRH data collection in humanitarian crisis situation [32] and scholarly literature [33]. However, selecting the appropriate sampling approach was challenging given the humanitarian context. Following the influx, a total of 4300 acres of hills and forests were cut down in Ukhiya and Teknaf sub-districts of Cox’s Bazar in order to accommodate the Rohingya refugees [34], to make their temporary shelters, and makeshift camps. Majority of these camps are located in remote locations in hilly landscapes that lack consistency in block and household arrangements and are overcrowded.

Initially, the team planned to follow random sampling at all stages for selecting survey respondents. However, the humanitarian setting was unknown to the research team and there was a very few researches conducted about the situation of SRH services and service seeking behavior among Rohingya refugees at the time when this research was conducted.. Therefore, there was less information available about possible feasible methodology for selecting appropriate sampling strategy. Hence, before finalizing the sampling strategy, the research team decided to familiarize themselves with the camp-setting. The team spent 1 month understanding the context and identifying suitable strategies by visiting several camps. They informally had discussions with community members and stakeholders to identify potential gatekeepers and challenges the research team may encounter during data collection. That exploration helped the team familiarize themselves with the settings, which ultimately helped in planning and logistical arrangements for data collection.

After familiarization, the research team decided to test the feasibility of random sampling strategy. While pre-testing, they found simple random sampling difficult due to the structure of the camps. The makeshift houses were built sporadically on hills with no identification numbers. Furthermore, the registration of the migrated FDMN people was happening during the data collection period which also created tensions among the refugee population. No complete list of FDMNs living in a specific block or camp was available and the registered FDMNs were also reluctant to share their registration numbers which created challenges in making a sampling frame. Due to the time constraint, it was also not possible to list down households for making sampling frame for simple random sampling. Thus, the research team decided to change their sampling strategy after the field test and chose systematic random sampling.

#### Strategies employed

We followed a multi-stage systematic sampling strategy - first selected camps randomly from the camp list, then lists of community leaders, named Majhii, of the selected camps were collected from the respective Camp In-Charge Offices. Afterwards, one Majhii from each selected camp was randomly selected. Then households were selected through systematic random sampling method. The selected Majhiis’ houses were marked as the center and then households in every ten footsteps from the left and right side were selected until the desired number of samples were reached. Researches that assess the SRHR among refugees in forced migration condition, multi-stage sampling techniques generally start by randomly selecting households and/or the units of living spaces through random sampling [20]. The modification in our sampling technique was to randomly select camps and Majhiis, before selecting the households. Mahjiis helped us to get the exact location of the blocks (their catchment areas) within the camps which were hard to locate at times, as they are spread out in a maze-like manner with many situated in the hills.

Maintaining the hierarchical administrative procedures for collecting camp and block-wise updated Majhiis-lists from the respective camp administration offices was a lengthy process. To effectively manage lengthy processing time of collecting Majhii’s list and complete data collection in time, the research team took help of stakeholders who were in-charge of camp management. The research team presented the research objectives and design in the regular SRHR sub-sector meeting with the stakeholders. Then the team communicated with the relevant organizations in-charge of the selected study camps before starting data collection and requested them to share the Majhiis-list of the respective camps. Following this strategy, the research team was able to collect Majhii’s lists beforehand and thus planned data collection accordingly. After collecting Majhii’s list and randomly selecting Majhii following the strategy mentioned above, the male members of the research team communicated with the selected Majhii over the phone and sometimes also visited them physically before starting data collection in the respective camp. They oriented selected Majhii about the research objectives and data collection plan, and also collected information about his/her catchment areas. This prior communication was helpful for rapport building with the community gatekeepers.

### Community trust related challenges

Gaining community’s trust is very important for any research [31]. However, the issue of trust between researchers and research participants is multi-layered and influenced by multiple determinants, especially in the humanitarian context which is a very complex and fragile situation [31]. People who are forcibly displaced from their community and forced to migrate in other countries for saving their lives are very vulnerable and traumatized [31, 35]. In addition, their position and identity in the host country, unsettled grievances, and political tension between home and host country also elevate the mistrust and suspicion among the refugee population [31]. The researchers faced similar challenges related to the trust of the Rohingya refugee community.

#### Challenges

The issue of FDMN is a very sensitive political issue for the Government of Bangladesh. The Government of Bangladesh has not recognized FDMNs as convention refugees. There is always political tensions between Bangladesh and Myanmar regarding the repatriation of the Rohingya community who fled to Bangladesh. Such political movements and decisions led to unrest and triggered the wariness among the FDMNs in some camps at different points of data collection. The tensions and rumors regarding the political steps of registering the FDMN population residing in the refugee camps created distrust among the FDMNs about the Bangladeshi people. Interestingly, during the informal discussions with some FDMNs the researchers found that FDMNs comparatively trust non-Bangladeshi nationals than Bangladeshi nationals and prefer to visit health centers operated by international organizations. According to them Bangladeshi nationals have conflict of interest regarding them staying in Bangladesh.

Therefore, researchers’ identity and positionality being citizens of Bangladesh put them under suspicion with the FDMNs, which ultimately made data collection more challenging in some camps. The FDMNs were supposed to be registered with an identification (ID) number, however, that was also incomplete or absent in many cases during data collection. When the research team tried to collect IDs by visiting individual households for making a sampling frame, FDMNs refused to show their registration cards as they feared this may lead to repatriation to Myanmar. They were suspicious about the intention of the researchers’ visit, and some of them initially refused to participate. This was not surprising given their past experiences in Myanmar, but also, they were in an unfamiliar environment, fully dependent and vulnerable to Bangladeshis and foreigners providing services. However, it was difficult to anticipate the challenges as those were not same across the camps. Even within the same camp, the situation was different in different days of data collection.

#### Strategies employed

The involvement of gatekeepers to access respondents was key. The research team identified that the local community leaders i.e. the Majhiis were instrumental for ensuring access, as well as providing detailed information on the communities’ overall cultural and religious beliefs and perceptions. Majhiis play the role of focal points for camp governance, channeling communication to the refugee community, handling small disputes and guaranteeing security [36]. This puts the Majhiis in a unique position of power dynamics within the refugee community. So, the research team established a relationship with the Majhiis. The team explained the purpose of the research and the importance of understanding women’s SRH needs and priorities to Majhiis and requested to convince the community people and the selected respondents to participate in the study. As the community trust Majhiis, when the Majhii introduced research team to the community, they agreed to cooperate and participate in the research. However, reaching those Majhiis through proper channels did not always guarantee their cooperation during data collection. In the specific camp sites where Majhiis were cooperative, it was comparatively easier to build rapport and gain trust. On the other hand, this strategy to gain access to respondents had limitations as well. In camps where Majhiis were not cooperative with the research team, they created barriers to data collection by not giving camp information, negatively influencing respondents to participate, etc.

In addition to involving Majhiis as the bridge to gain trust of the community, research team also tried to link the individual respondents with appropriate referral networks. After completion of an interview, the female members of the research team also gave SRH-related health service availability information (within the camp) to the respondent. When the research team identified any respondent needed medical and psychosocial support, they informed the respondent from where they can seek support and. The researchers also informed the relevant stakeholders about the respondent and the support she/her family requires. This safeguarding strategy also helped research team to gradually gain trust with individuals in some cases.

## Conclusions

While conducting SRH-related research in a humanitarian context, it is crucial to have proper understanding about the context and the community before even designing the research. The lessons learned from this research will be very useful for sensitizing future researchers investigating sensitive topics like SRH of refugee populations. Contextual adaptation of research methods and involving community and local key stakeholders in data collection are the key lessons learnt from this study. It was an important learning for the research team to be innovative with the methodologies in humanitarian context research Another key lesson was the identity and positionality of researchers as a member of the host country may create distrust and suspicion among the refugee populations, which may make them reluctant to cooperate with the research team. The multi-level complexities of humanitarian settings may introduce unforeseen challenges and interrupt research plans. Timely and contextual adaptations are crucial to address the challenges induced at different stages of research in humanitarian context.

## Data Availability

Not applicable.

## Abbreviations

SRHR: Sexual and Reproductive Health and Rights
SRH: Sexual and Reproductive Health
FDMNs: Forcibly Displaced Myanmar Nationals
CEmOC: Comprehensive Emergency Obstetric and newborn Care
ANC: Ante Natal Care
FP: Family Planning
MISP: Minimum Initial Service Package
IDIs: In-depth Interviews
KIIs: Key Informant Interviews
FGD: Focus Group Discussion
INGO: International non-government organization
NGO: Non-government organization
WHO: World Health Organization
UNFPA: United Nations Population Fund
PHC: Primary Health Center
RRRC: Refugee Relief and Repatriation Commission
CIC: Camp In-Charge

## Footnotes

1 **Collective site** refers to camp-like settings where only Rohingya refugees live. This category encompasses the previous Formal Refugee Camps, Makeshift Settlements and part of those Spontaneous Settlements where no Bangladeshi communities live.

2 **Collective site with host community** refers to those collective camp-like settlements that developed around existing Bangladeshi communities, and hence present a mixed population.

3 **Dispersed site in host community** refers to villages and dispersed locations where Rohingya refugees reside among Bangladeshi host communities.

4 **Majhiis** are local leaders selected by Bangladesh Army and the Camp In-charge (CIC) offices from the Rohingya refugees, who are responsible for managing some administrative issues (reaching refugees, relief distribution, etc.) for a certain number of households in a designated area.

5 **The Minimum Initial Service Package (MISP)** for SRH, developed by the Inter-agency Working Group (IAWG) on Reproductive Health in Crisis, is a standard set of priority activities including emergency obstetric care, prevention and treatment of STI/HIV, clinical management of rape, and referrals, which needs to be implemented immediately after the onset of a humanitarian crisis situation.

6 **The comprehensive SRH service package** includes family planning; antenatal, intrapartum, postpartum and newborn care; cervical cancer screening, menstrual regulation (MR); and referrals in addition to the services under MISP.
