# Publisher Correction to: Challenges and strategies in conducting sexual and reproductive health research among Rohingya refugees in Cox’s Bazar, Bangladesh

**DOI:** 10.1186/s13031-020-00335-4

**Published:** 2020-12-30

**Authors:** Rushdia Ahmed, Bachera Aktar, Nadia Farnaz, Pushpita Ray, Abdul Awal, Raafat Hassan, Sharid Bin Shafique, Md Tanvir Hasan, Zahidul Quayyum, Mohira Babaeva Jafarovna, Loulou Hassan Kobeissi, Khalid El Tahir, Balwinder Singh Chawla, Sabina Faiz Rashid

**Affiliations:** 1 BRAC James P Grant School of Public Health, BRAC University, 5th Floor, (Level-6), icddrb Building, 68, Shaheed Tajuddin Ahmed Sarani, Mohakhali, Dhaka, 1212 Bangladesh; 2 Department of Reproductive Health Research, World Health Organization, Geneva, Switzerland; 3 Health Sector Coordination Office, World Health Organization, Cox’s Bazar, Bangladesh

**Correction to: Confl Health 14, 83 (2020)**

**https://doi.org/10.1186/s13031-020-00329-2**

In the publication of the original article [1] an error was introduced during the typesetting process. Due to this: the original publication did not show the correct equal contribution of the author groups.

In this correction article the correct and incorrect information is shown. The original article has been updated.

**Incorrect:**

† Rushdia Ahmed, Bachera Aktar, Nadia Farnaz, Pushpita Ray, Abdul Awal, Raafat Hassan and Sharid Bin Shafique contributed equally to this work.

**Correct:**

† Rushdia Ahmed, Bachera Aktar and Nadia Farnaz contributed equally to this work.

† Pushpita Ray, Abdul Awal, Raafat Hassan and Sharid Bin Shafique contributed equally to this work.
